# Centennial of Pyloromyotomy

**Published:** 2013-01-01

**Authors:** V Raveenthiran

**Affiliations:** Department of Pediatric Surgery, SRM Medical College and Hospital SRM University, Chennai, India.

 (Athena stands for abbreviation of Abstracting and Thoughtful Evaluation of Neonatal Articles; but it is also personified by the contributor. Like Athena of Greek mythology, she distills wisdom from published literature)

Shelf-life of scientific discoveries is usually less than few decades because science itself is an ever evolving discipline. Therefore, it is truly remarkable that pyloromyotomy described by Conrad Ramstedt (Fig. 1) is celebrating centennial this year. In October 1912, Ramstedt described the cure of hypertrophic pyloric stenosis (HPS) and that serendipitous discovery has stood the test of 100 years! (Fig. 2) It is also the most satisfying of all surgical procedures that offers consistent cure and it has saved millions of infants from premature death. Athena is pleased to join the fraternity of pediatric surgery in celebrating Ramstedt’s achievement [1]. 

Colossal success of a curative procedure usually obviates the need for further scientific research by solving the underlying problem. Athena is curious to know the impact of Ramstedt on the research and literature pertaining to HPS. She searched Pubmed titles using the key words “pyloromyotomy”, or “hypertrophic pyloric stenosis”. Contrary to the expectations, the number of research articles has progressively increased over the last century (Fig. 3). Some of the recent research papers conclude diametrically opposite of the views held in the past.

**Figure:**
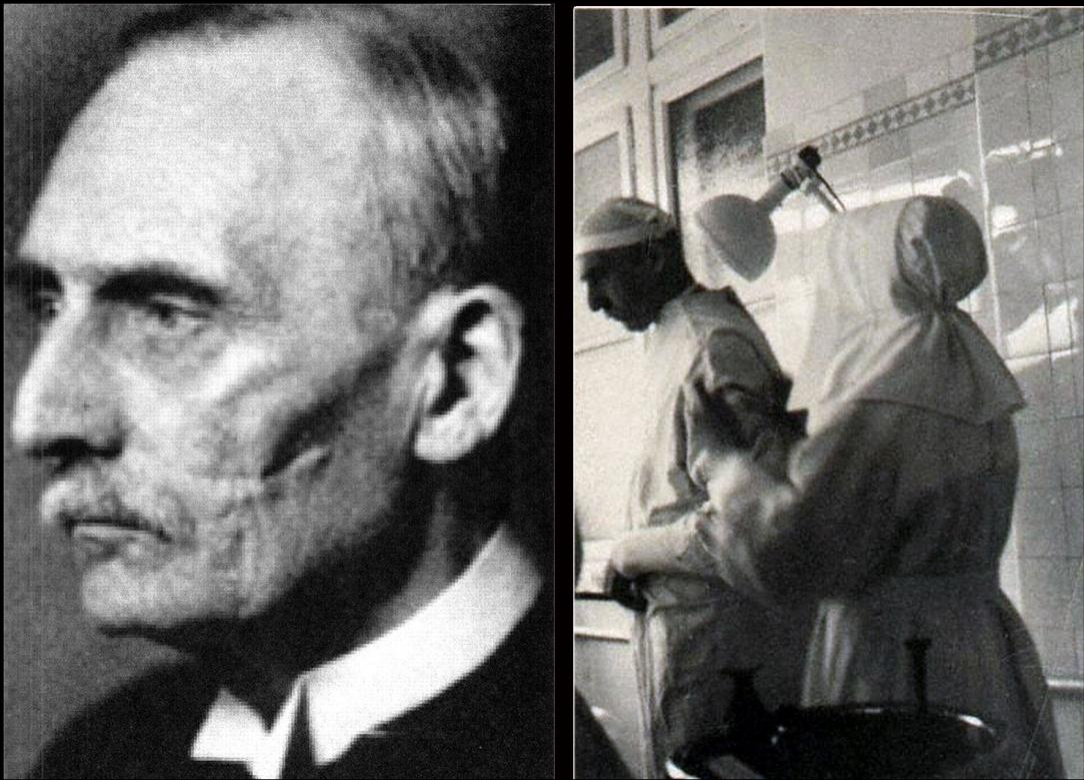
Figure 1: Profile photograph of Conrad Ramstedt (Left). Professor Ramstedt is getting ready to perform a pyloromyotomy (Right). (Original source of these photographs and copyright status are unknown - Reproduced under “Fair use” doctrine for non-profit academic purpose).

**Figure:**
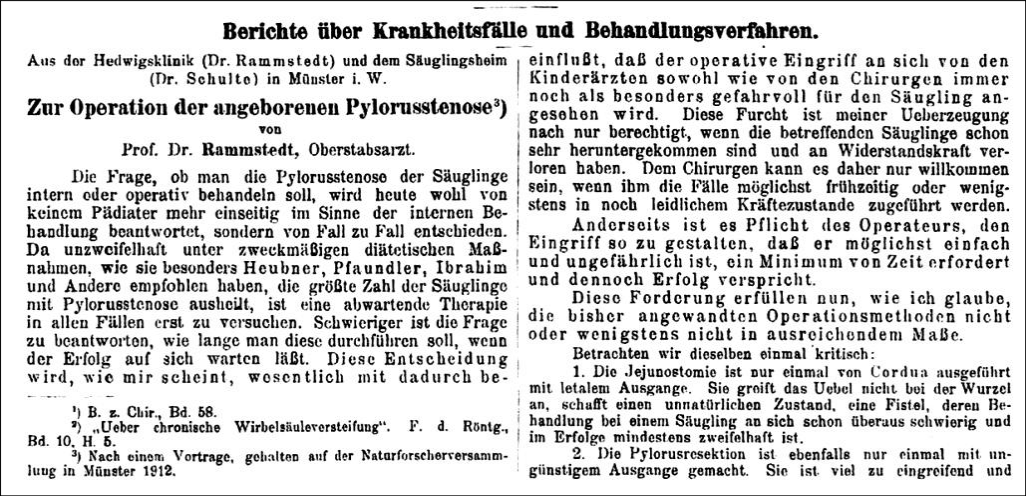
Figure 2: Title page of the historic article.

**Figure:**
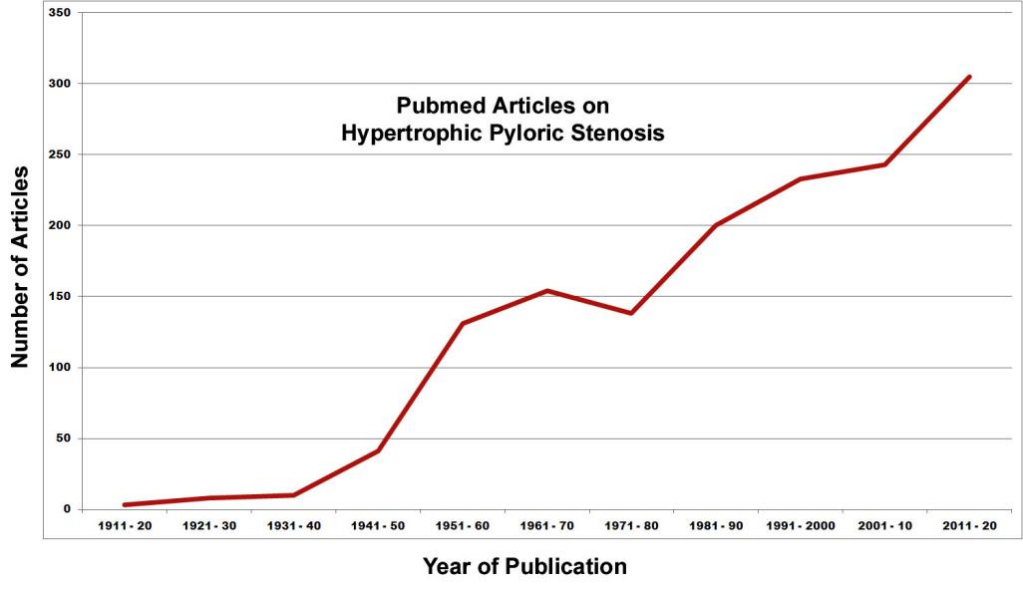
Figure 3: Number of Pubmed-indexed articles on Hypertrophic pyloric stenosis published during the last 100 years. (Plots for 2011 -20 period were calculated by projecting the number of articles published during 2011-12).

Contrary to the endurance of Ramstedt’s operation, diagnostic modality has drastically changed in the last few decades. The younger generation of pediatric surgeons, probably, no longer has the tact and patience of palpating the “pyloric olive” - “the peanut under the blanket”. [2] Increasingly more reliance is placed on imaging studies. Conventionally, pyloric muscle thickness more than 3 mm in ultrasonography is considered diagnostic of HPS. The rationale of this arbitrary number has recently been questioned. This criterion is promulgated based on the common age of clinical presentation at around 3 to 6 weeks. However, this rigid criterion is likely to miss HPS in its evolving phase prior to 3 weeks. Illogicality of it is also obvious in preterm infants in whom the muscle mass is expectedly thinner than term neonates. Pyloric muscle under physiological contraction or spasm may appear deceptively thick. Therefore, several workers have recently attempted to rationalize the diagnostic criterion.

Iqbal et al [3] have studied the correlation between thickness and length of the pylorus with age and weight of the infant. They used linear 12 MHz transducer to study 304 infants of whom 67 had proven HPS. Initial scan was reported as negative in two HPS because of muscle thickness less than 3 mm. But a repeat scan after 5 days showed a muscle thickness of 3.3 mm and 4.9 mm respectively. In one patient HPS was diagnosed despite muscle thickness (2.9 mm) falling short of the diagnostic criterion. They have shown, using Pearson correlation, a negative correlation between pyloric thickness versus age and weight in healthy infants (i.e. muscle thickness decreases with increasing age or weight). Contrarily, the correlation was positive in HPS infants. Athena is enthralled, yet puzzled, by the observation that the pyloric muscle undergoes ontogenic regression in thickness with increasing age. Understanding more about this phenomenon may divulge the pathogenic secrets of HPS. However, she is also disappointed that the sample size of the study is too small to form age or weight specific nomograms. In a similar study involving 189 proven cases of HPS, Said et al [4] showed that muscle thickness positively correlates with age and weight while pyloric length has no correlation with these parameters.

Huang et al [5] examined if pyloric ratio is better than linear measurements in a small series of 12 infants. Although a diagnostic ratio between pyloric wall thickness and pyloric diameter was described by Lowe et al in 1999, the concept is yet to find its acceptance in clinical practice. Huang et al [5] have proposed a new ratio called Alternative Pyloric Ratio (APR). It is defined as the ratio of pyloric intermuscular space diameter to the diameter of the pylorus. Lowe’s ratio more than 0.30 or APR less than 0.11 are diagnostic of HPS. However, APR was better than Lowe’s ratio in predicting adequacy of pyloromyotomy in follow-up scans. With adequate myotomy, ARP doubled as early as the first post-operation day while Lowe’s ratio remained unchanged for several days. Every pediatric surgeon might have experienced an occasional case wherein vomiting recurs after pyloromyotomy. The usual dilemma is about the adequacy of myotomy and the need of a reoperation. Athena finds the concept of APR very useful in such situations. If APR has doubled in the follow-up sonography, post-operative vomiting is unlikely to be due to inadequate myotomy. 

Etiology of HPS is a fascinating subject. Adding to a long list of etiologic factors Krogh et al [6] recently implicated bottle-feeding. They studied 70148 singleton infants using Danish National Birth registry. There were 65 infants with HPS of whom 36 had exclusive breastfeeding and 29 had bottle feeds. Hazards ratio was calculated using a Cox regression model. The authors conclude that the risk of HPS in bottle-fed infants was 4.6 fold higher than that of breastfed infants. Two decades ago textbooks used to mention breastfeeding as a risk factor of HPS [7]. Athena is amused by the swing of pendulum in the opposite direction. At the same time she did not fail to note that Krogh et al were conveniently silent as to the feeding practices of the remaining 70083 infants who did not develop HPS. How many of the 70083 infants were bottle-fed and why did they not develop HPS? Sensibleness perhaps counts more than statistics. Non-bilious projectile vomiting is traditionally considered characteristic of HPS. Because of the physical occlusion of pyloric canal, bile-stained vomiting is logically impossible in HPS. But, medicine is a soft science defying all logics. In a retrospective cohort of 354 HPS Piroutek et al [8] have shown bile-stained vomiting in 5 (1.5%) infants. Interestingly, pyloric muscle thickness was smaller in them than those who did not have bilious vomiting. Athena is surmising as to the nature of electrolyte and acid-base imbalance occurring in this subset of HPS with bilious vomiting. 

Simplicity of approach, absence of complicated dissections and suturing maneuvers, consistent cure and short duration of surgery are characteristic of Ramstedt’s operation. These attributes have made pyloromyotomy an ideal tool for testing newer modalities of minimally invasive surgery. In the last 10 years there have been 4 meta-analyses [9 - 12] comparing laparoscopic pyloromyotomy (LP) versus open pyloromyotomy (OP) [Table 1]. Another randomized controlled trial (RCT) [13] published subsequent to these meta-analyses showed no significant difference between OP versus LP with respect to complications (7.1% vs. 3.6%), operation time (52 vs. 55 min), time of re-feeding (6.04 vs. 6.00 hrs) and length of hospital stay (1.1 vs. 1.3 days). Body image scores (28 vs. 24) and cosmetic score (27 vs. 17) were significantly higher in LP than OP. Athena, after carefully considering all these evidences, is not convinced about the superiority of LP over OP. Not infrequently meta-analyses and RCTs conclude in favour of LP based on few hours of difference in the time of re-feeding or hospital stay. Athena wish to remind that statistical significance need not be synonymous with clinical significance.

**Figure:**
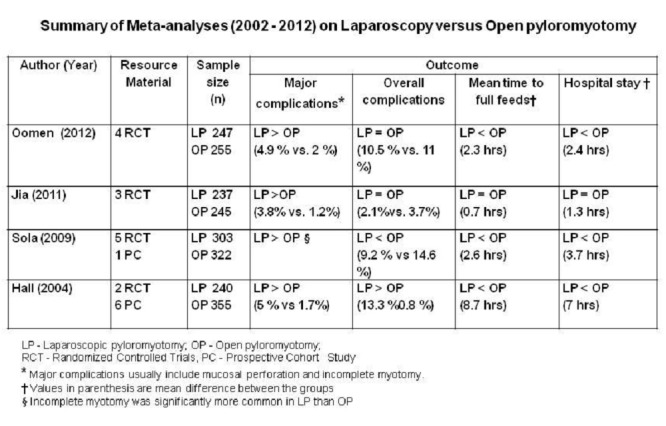
Table 1: Summary of Meta-analyses (2002 - 2012) on Laparoscopy versus Open pyloromyotomy

Furthering the concept of minimally invasive surgery, Turial et al [14] have described a micro-laparoscopic technique. Instead of conventional laparoscopic instruments they have used specially designed - needle-like - 2 mm instruments. This technique has also been referred to as needle-scope surgery. In a prospective cohort of 110 HPS they compared micro-laparoscopic pyloromyotomy (MLP; n=28) with Bianchi’s circumumbilical pyloromyotomy (BUP; n=56) and conventional open pyloromyotomy (COP; n=26) [15]. MLP was found to be advantageous over BUP and COP with respect to operating time (20 vs. 38 vs. 50 min), time of re-feeding (32 vs. 48 vs. 70 hrs) and hospital stay (82 vs. 75 vs. 90 hrs). There were no major complications in any of the groups. Recent enthusiasm on single-incision laparoscopy (SILS) is yet another advancement in minimal invasive surgery. In this method, a single umbilical port is used for instrumentation instead of the 3 or 2 ports of conventional laparoscopy. Kozlov et al [16] compared 24 LP and 12 SILS pyloromyotomies. Operating time, time of re-feeding, analgesic requirement and hospital stay did not significantly differ between the groups. As a variant of the theme, Bertozi et al [17] reported laparoscopy-assisted single incision pyloromyotomy in 19 infants. In this hybrid procedure, pylorus is visualized using a telescope inserted through a single umbilical port. Under laparoscopic vision the pyloric olive is grasped and exteriorized through the umbilical-port incision. Pyloromyotomy is then completed by conventional open technique. 

Crowding of instruments in SILS makes it technically demanding. In conventional laparoscopy, grasping instrument held in surgeon’s left hand is used to retract the first part of duodenum while the right hand instrument is used to perform the myotomy. But in SILS such a maneuver is ergonomically cumbersome, if not impossible. Harmon’s team has described a “cross-technique” to circumvent this problem [18, 19]. In this technique, surgeon’s left hand instrument is used to grasp the antrum of stomach and traction is applied leftward to expose the pylorus diagonally. Right hand instrument is used as usually to carryout myotomy. Thus, the two instruments are crossed intra-abdominally - a concept alien to laparoscopic surgery. 

Athena wonders at the insatiable desire of man to avoid scars. After familiarizing laparoscopy, micro-laparoscopy and SILS, the pagan has now moved to a new deity called NOTES (Natural Orifice Transluminal Endoscopic surgery). Kawai et al [20] have recently described a novel technique of endoscopic pyloromyotomy. In this technique a flexible endoscope is passed per orally into the stomach and saline is injected into the submucosa of pylorus. Through a tiny mucosal incision, a submucosal tunnel of 5 cm is created. Visualizing through this tunnel, myotomy of circular fibers is done until the outer longitudinal muscle layer is reached. Myotomy is stopped at this point and the mucosal incision is re-approximated using endoscopic clips. Feasibility of this technique was tested in a porcine model. Pyloric resting pressure dropped from 16 mmHg to 6 mmHg immediately after the procedure. There were no acute complications such as bleeding or perforation. Although technical feasibility of endoscopic pyloromyotomy has been established its safety and applicability in human infants is yet to be ascertained. Athena keeps wondering as to how many more ways are there in store to skin the proverbial cat.

Dramatic relief of symptoms following pyloromyotomy has lured many pediatric surgeons to declare a cure after one or two follow-up visits. Long term results are generally presumed to be good rather than proved by evidences. Two recent papers question this common assumption. Using Baylay scales, Walker et al [21] assessed neurological development of 52 infants with HPS and 211 healthy infants at 1 year of age. Cognitive, receptive language and motor scores were significantly lower in HPS infants than in controls. It is unclear whether this adverse outcome is attributable to the disease process or to anesthesia administered during surgery. Although further studies are needed to conclude whether this difference is of any practical significance, the findings of this study are certainly a source of concern. Saps and Bonilla [22], in a case-control study, studied 100 HPS patients and 91 controls in their late childhood. The mean follow-up period was 7.2 years. Nearly 25% of those who underwent pyloromyotomy in infancy developed chronic abdominal pain at a later age; while only 6% of the controls were so. Irritable bowel syndrome, functional dyspepsia and functional abdominal pain were more common in HPS group than in control group. Athena considers these two studies as eye-openers necessitating long-term follow-up of HPS infants.

## Footnotes

**Source of Support:** Nil

**Conflict of Interest:** The author is Editor of the journal. But he did not take part in the evaluation or decision making of this manuscript. The manuscript has been independently handled by two other editors.
